# Identification of Enantiomeric Byproducts During Microalgae-Mediated Transformation of Metoprolol by MS/MS Spectrum Based Networking

**DOI:** 10.3389/fmicb.2018.02115

**Published:** 2018-09-07

**Authors:** Min Lv, Ching Lo, Cheng-Chih Hsu, Yuwen Wang, Yin-Ru Chiang, Qian Sun, Yang Wu, Yan Li, Lingxin Chen, Chang-Ping Yu

**Affiliations:** ^1^CAS Key Laboratory of Coastal Environmental Processes and Ecological Remediation, Yantai Institute of Coastal Zone Research, Chinese Academy of Sciences, Yantai, China; ^2^Graduate Institute of Environmental Engineering, National Taiwan University, Taipei, Taiwan; ^3^Department of Chemistry, National Taiwan University, Taipei, Taiwan; ^4^CAS Key Laboratory of Urban Pollutant Conversion, Institute of Urban Environment, Chinese Academy of Sciences, Xiamen, China; ^5^Biodiversity Research Center, Academia Sinica, Taipei, Taiwan

**Keywords:** metoprolol (MPL), microalgae, enantioselective biodegradation, transformation products, biodegradation pathway, MS/MS spectral similarity networking

## Abstract

Metoprolol (MPL) is a chiral β-blocker ubiquitously detected in various environments due to its low to moderate removal in wastewater treatment plants. This study was conducted to test the potential of using microalgae to degrade emerging contaminant MPL and to characterize the enantiomeric enrichment during MPL degradation by microalgae. The results showed that PO_4_^3−^- P, NO_3_^−^- N and MPL could be simultaneously removed in the synthetic effluent by the targeted microalgae species, indicating microalgae were promising in wastewater treatment. Stereoselectivity was observed during MPL degradation by microalgae, with R-form enrichment. A marginal linear relationship between MPL degradation and enantiomeric enrichment was observed, implying that the enantiomeric tool, used as a quantitative indicator of biodegradation, could possibly be applied in MPL degradation by microalgae. An efficient liquid chromatograph tandem high resolution mass spectrometry (LC-HRMS/MS) chiral analytical method was developed to identify transformation products (TPs). The results showed that MS/MS spectral similarity networking could be used as a powerful tool to quickly identify unknown TPs. A total of 6 pairs of chiral TPs were identified, among which two new TPs of MPL including hydroxy{4-[2-hydroxy-3-(isopropylamino)propoxy]phenyl}acetic acid (α-HMPLA) and 4-[2-Hydroxy-3-(isopropylamino)propoxy]benzaldehyde (DMPLD) were firstly reported, and proposed transformation pathways of MPL by microalgae were given. Considering the paired TPs detected and that the degradation of the two enantiomers followed first order kinetics, the two enantiomers likely had the same degradation mechanism.

## Introduction

The occurrence of pharmaceuticals in the wastewater is an issue of great concern, because current wastewater treatment processes can not effectively remove them (Sun et al., 2014; Ashfaq et al., 2017; Ben et al., 2018). Therefore, residual pharmaceuticals in the wastewater treatment plant effluent are discharged into the receiving water, leading to the ubiquitous detection of pharmaceuticals in the aquatic environment worldwide (Lv et al., 2014; Sun et al., 2016; Tarpani and Azapagic, 2018; Wilkinson et al., 2018). With the increasing concern of the potential risks which pharmaceuticals could pose to ecology and human health, enhanced removals of pharmaceuticals in wastewater treatment systems are increasingly needed.

Microalgae have been suggested to be promising in wastewater treatment, since it could efficiently “recycle” nitrogen (N) and phosphorus (P) from treated effluents (Menger-Krug et al., 2012; Cai et al., 2013; Van Wagenen et al., 2015). Meanwhile, some researchers found that some microalgae species showed good removal of emerging contaminants such as pharmaceuticals (Bai and Acharya, 2017) and endocrine disrupting compounds (Sole and Matamoros, 2016). Therefore, there is a great potential of applying microalgae in treated effluent wastewater. However, considering limited microalgae species and pharmaceuticals have been tested, there is a need to examine more algae-mediated removal of pharmaceuticals and to elucidate pathways for algae-mediated pharmaceutical transformation.

It is worth noting that at least 50% of pharmaceuticals have their chirality and it has been overlooked by researchers studying the fate of chiral compounds in the environment. Many of the chiral pharmaceuticals are marketed as racemates with two enantiomers (Nikolai et al., 2006). Enantiomers of chiral pharmaceuticals can have stereospecific interactions with biological systems despite their similar physicochemical properties. Therefore, the enantiomeric ratio could be changed after stereospecific interactions. Additionally, due to different biological activity of enantiomers, the same pharmaceuticals might reveal different activity and toxicity. For example, it has been demonstrated that the S form of fluoxetine is 9.4 times more toxic than R form to *Pimephales promelas* (Stanley et al., 2007). However, despite the significance of the role which chirality plays in the fate and toxicity of pharmaceuticals, chirality has not been given adequate attention for consideration of the degradation of chiral pharmaceuticals in the environment.

Among chiral pharmaceuticals, metoprolol (MPL), widely used to treat cardiovascular diseases, has a high consumption (Dong et al., 2013) and low elimination rate in wastewater treatment plants (Maurer et al., 2007; Scheurer et al., 2010; Sun et al., 2014). Therefore, it is of great importance to explore the possibility of using microalgae to removal MPL. However, till now, information concerning the removal of MPL by the microalgae is still limited, not to mention the transformation pathway by the microalgae. Additionally, enantiomeric fraction (EF) of enantiomers is critical to evaluate their susceptibility to biodegradation, and has recently been suggested as predictive tools to elucidate the fate of chiral pharmaceuticals (Jammer et al., 2014; Souchier et al., 2016). For instance, a linear relationship was obtained between aerobic biodegradation of MPL and enantiomeric enrichment in wastewater treatment plant through the application of Rayleigh equation (Souchier et al., 2016). However, inconsistent results were also reported by Matamoros et al. (2009), who found that the EF value of ibuprofen was not correlated with its removal efficiency due to different enantioselective degradation kinetics under aerobic and anaerobic conditions in the wastewater treatment plant (Matamoros et al., 2009). Besides, different results with chiral MPL, either S-form enrichment (Evans et al., 2017) or R-form enrichment (Ribeiro et al., 2013) in environment, have been observed. These results suggested that since the environmental processes affecting enantioselective fate of contaminants were complex, different biodegradation processes including biodegradation by microalgae should be considered. However, investigation on enantioselective fate of MPL by microalgae degradation has not been reported yet.

Considering these concerns, this study aims to discuss the enantiomer-specific degradation of chiral MPL by different microalgae species in synthetic effluent, and elucidate the transformation pathways. Results from this study are expected to help ascertain stereoselectivity of chiral MPL during degradation by microalgae, and improve our understanding of the behavior and fate of chiral MPL.

## Materials and Methods

### Materials and Chemicals

Racemic MPL (as tartrate) was purchased from Sigma–Aldrich (United States). (S) -MPL and (R) -MPL were obtained from Toronto Research Chemicals (Canada). Methanol and acetone (HPLC grade) were provided by Merck (Germany). Reagent water was prepared with a Milli-Q water purification system (Millipore, United States). Stock solutions of MPL were prepared in methanol and stored in −20°C in the dark. All other reagents were at least of analytical grade from commercial sources.

Four common microalgae species including *Selenastrum capricornutum*, *Chlorella vulgaris*, *Scenedesmus quadricauda* Breb and *Haematococcus pluvialis* were purchased from Institute of Hydrobiology, Chinese Academy of Sciences. The microalgae were grown in BG11 medium (Stanier et al., 1971) under autotrophic conditions at 25°C in a controlled-growth chamber (PGX-350B, SAFU Experimental Apparatus Technology) with a maximum light intensity of 12000 lux. We set up light intensity changes (12 h light/12 h dark cycle) using the following sequence: 20% maximum for 4 h, 40% maximum for 4 h, 20% maximum for 4 h, dark for 12 h, corresponding to 2400 lux for 4 h, 4800 lux for 4 h, 2400 lux for 4 h, 0 lux for 12 h, to simulate daily light exposure.

### Degradation of MPL by Microalgae in the Synthetic Effluent

To investigate the enantiomeric degradation of MPL, the microalgae, after reaching mid-logarithmic growth phase, were centrifuged from the growth culture, washed twice using the synthetic effluent (Xu et al., 2014) (see detailed composition in **Supplementary Table S1**), and then inoculated into 200 mL of synthetic effluent spiked with 5 mg L^−1^ MPL in 1 L flasks. This MPL concentration was used to explore the enantiomeric characterization and biodegradation pathways by microalgae. The initial microalgae number was 5 × 10^6^ cells/mL. Half of the flasks were autoclaved and used as control to investigate the abiotic degradation of MPL. All the experiments were undertaken in triplicate in the controlled-growth chamber under the conditions described above. Four milliliters of samples were taken at 0, 10, 19, 25, 35, and 40 day for microalgae growth observation, as well as water quality and MPL analysis. The number of microalgae was obtained by the microscope at 400X (Zeiss Axio Imager A1). The water quality parameters including PO_4_^3−^- P, NO_3_^−^- N and NH_4_^+^ were measured according to standard methods with detailed information provided in SI.

For MPL analysis, samples were centrifuged at 15000 rpm for 30 min. Liquid chromatography (UltiMate 3000, Dionex) with UV detection were used to analyze the samples. The injection volume was 10 μL and the detection wavelength was 226 nm. Separation of the enantiomers was performed using an Astec Chirobiotic^TM^ T column (250 mm × 4.6 mm, 5 μm) from Supelco and a binary gradient with a flow rate of 1.0 mL/min. An isocratic mode of elution consisting of 30 mmol/L ammonia acetate in water/methanol (10/90, v/v) was used. The elution order for MPL enantiomers was established by enantiopure standards, with (S)-MPL eluted before (R)-MPL (**Supplementary Figure S1**).

For enantiomeric enrichment determination, EF was calculated with Equation (1) (Bagnall et al., 2012), where E1 and E2 are the concentrations of the first and the last eluting enatiomer. For racemate metoprolol, the EF value is 0.5.

(1) $$\text{EF}=\frac{\text{E}1}{\text{E}1+\text{E}2}$$

Enantiomer enrichment factor (ε_ER_) values were determined using the Rayleigh equation [Equation (2)] (Jammer et al., 2014).

(2) $$\ln(\frac{{\text{ER}}_{\text{t}}}{{\text{ER}}_{0}})={\text{ε}}_{\text{ER}}\times \ln f$$

Where ER_t_ and ER_0_ are the current and initial ratio between the two enantiomers and *f* is the compound remaining fraction (C_t_/C_0_).

### Identification of Transformation Products

For identification of transformation products (TPs), 10 mL samples during degradation process were prepared using a solid phase extraction (SPE) /purification step according to previous method (Sun et al., 2014) with detailed information provided in SI. Liquid chromatography tandem high resolution mass spectrometry (LC-HRMS/MS) in positive ion mode using an Orbitrap Elite^TM^ Hybrid Ion Trap-Orbitrap Mass Spectrometer (Thermo Fisher Scientific, United States) was used to analyze the prepared samples. The separation method was described as in the previous section. For MS acquisition, a data-dependent acquisition was performed to obtain the comprehensive metabolomic dataset. In short, a reference scan of MS1 spectrum were acquired by orbitrap at a resolution of 60,000 and the *m/z* range of 150–1300 to determine the top 10 abundant precursor ion *m/z*. Subsequently, the fragment ion scans were continued in the linear ion trap of the hybrid MS with the top 10 abundant precursor ions using collision-induced dissociation (CID) at 30 normalization energy. All the data were converted to mzXML format using msconvert (Chambers et al., 2012) and uploaded to the Global Natural Products Social Molecular Networking (GNPS)^1^.

Global Natural Products Social Molecular Networking has been used for the elucidation of microbe-derived compounds by spectral similarity alignment, which provides an efficient way to categorize structurally related molecules (Nguyen et al., 2013; Wang et al., 2016). We assumed that TPs of MPL were structurally similar to MPL, and therefore TPs and MPL should share similar MS/MS spectra. GNPS was thus used in this study to explore its applicability in identification of TPs. The MS/MS spectral similarity networking using GNPS was conducted as previously described (Wang et al., 2016). Briefly, the data was filtered by removing all MS/MS peaks within ±17 Da of the precursor m/z. The identical MS/MS spectra was then merged together (with parent ion and fragment ion tolerance of 0.003 Da) to create consensus spectra. The molecular network was then created using a cosine similarity threshold of 0.6.

### Statistical Analysis

One-way ANOVA was conducted to determine whether there were significant differences in the values. A difference was considered significant when *p* < 0.05. The statistical analyses were performed using SPSS 16.0

## Results and Discussion

### Assessment of Nutrient and Metoprolol Removal Efficiency by the Microalgae in Synthetic Effluent

**FIGURE 1:**
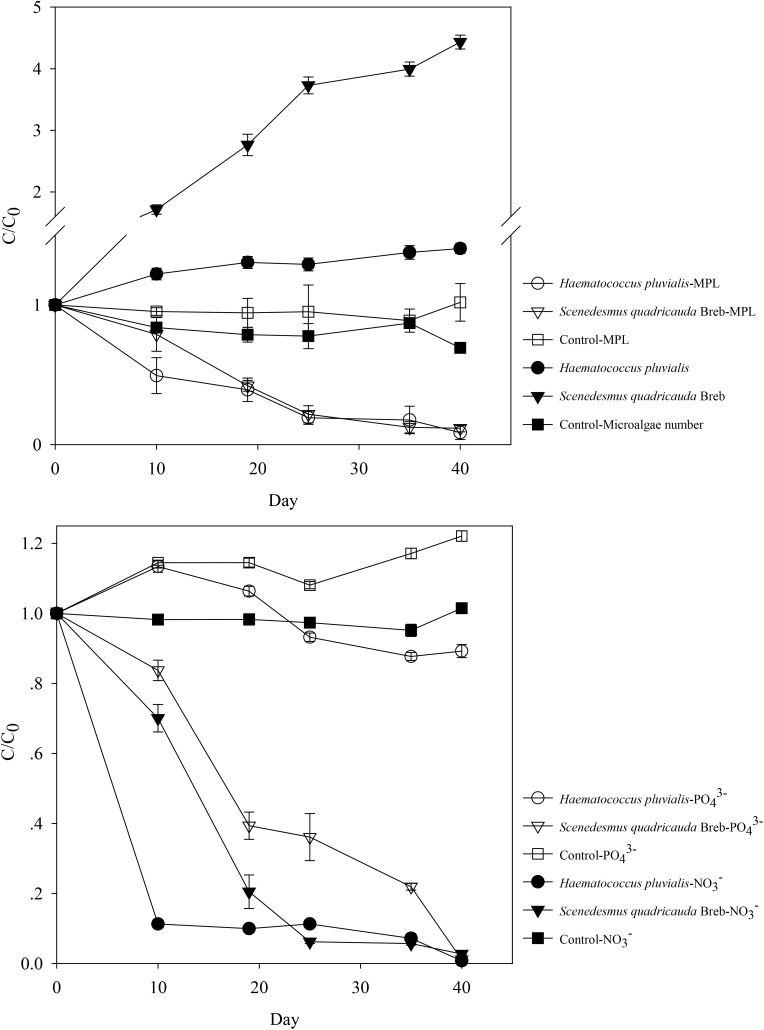
The removal of nutrient and metoprolol by *Haematococcus pluvialis* and *Scenedesmus quadricauda* and their growth in the synthetic effluent.

The initial PO_4_^3−^- P and NO_3_^−^- N concentrations in synthetic effluent of treated wastewater were 5.89 and 12.04 mg L^−1^, respectively. As shown in **Figure 1**, *Haematococcus pluvialis* and *Scenedesmus quadricauda* could effectively remove NO_3_^−^- N and MPL in the synthetic effluent, with removal efficiencies of 99.1 and 97.3% for NO_3_^−^- N, and 91.4 and 90.7% for MPL, respectively after 40 days incubation. However, varied removal of PO_4_^3−^- P was observed with only 10.8% for *Haematococcus pluvialis* and 98.9% for *Scenedesmus quadricauda*. In the meantime, the microalgae growth was observed with the microalgae numbers increase by 1.4 fold and 4.4 fold for *Haematococcus pluvialis* and *Scenedesmus quadricauda*, respectively, which was in consistent with the higher removal of PO_4_^3−^- P by *Scenedesmus quadricauda*. For MPL, abiotic processes such as photolysis could be neglected in this study, since there was no significant removal in the control (*P* > 0.05), indicating that MPL removal was likely attributed to biodegradation. For both *Haematococcus pluvialis* and *Scenedesmus quadricauda*, the degradation of MPL followed first order kinetics with *R*^2^ above 0.95. In comparison with *Haematococcus pluvialis* and *Scenedesmus quadricauda*, the other two microalgae species could also efficiently remove PO_4_^3−^- P and NO_3_^−^- N in the synthetic effluent, while their removal rates of MPL were much lower (**Supplementary Figure S2**), implying the involvement of different mechanisms to remove MPL among different microalgae species. The results indicated that microalgae mediated MPL removal could be a potential strategy although the degradation rate could be slower since the effluent of treated wastewater might not be the optimized medium for alga growth.

### Enantiomeric Characterization of Metoprolol Degradation

**FIGURE 2:**
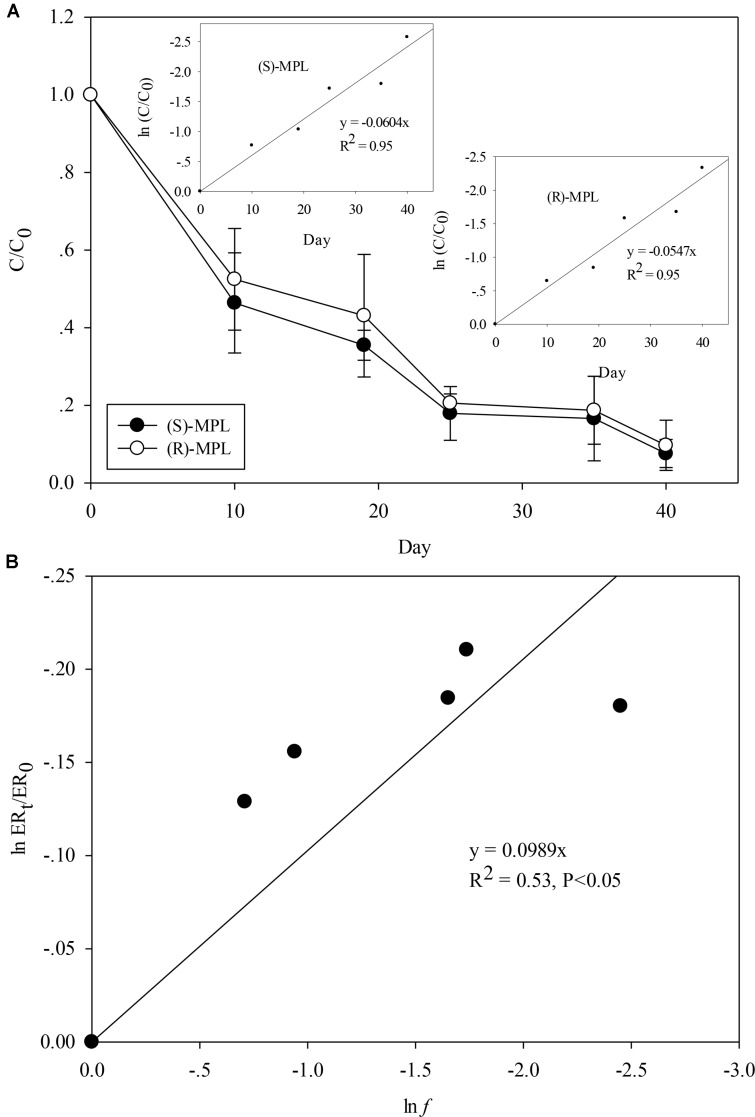
(S)-MPL and (R)-MPL concentrations evolution against time during MPL biodegradation by *Haematococcus pluvialis* **(A)** and Rayleigh representation of the enantiomeric enrichment of MPL versus MPL biodegradation **(B)**. Insets: Kinetics of first-order biodegradation of the two MPL enantiomers.

Recent study in environmental studies have obtained varied results showing (S)-MPL enrichment of MPL (Evans et al., 2017) and (R)-MPL enrichment of MPL (Ribeiro et al., 2013), highlighting the necessity to investigate the enantioselective fate of MPL during biodegradation. Considering that *Haematococcus pluvialis* and *Scenedesmus quadricauda* showed efficient removal for MPL, they were chosen for further enantiomeric characterization. To the best of our knowledge, this is the first report on enantiomeric characterization of MPL by the microalgae. As shown in **Figure 2A** and **Supplementary Figure S3a**, MPL biodegradation by *Haematococcus pluvialis* and *Scenedesmus quadricauda* was enantioselective with (S)-MPL being degraded faster than (R)-MPL, which was supported by the significant decrease (*p* < 0.05) in EF values during biodegradation (**Supplementary Figure S4**), resulting in the enrichment of (R)-MPL. Both the two enantiomers’ biodegradation curves fitted first order kinetics. Rate constants were found to be 0.0604 and 0.0547 d^−1^ for *Haematococcus pluvialis* and 0.074 and 0.0586 d^−1^ for *Scenedesmus quadricauda*, respectively.

Enantiomeric enrichment dependence of MPL on MPL biodegradation was also studied to investigate if the enantiomeric tool could be used as a quantitative indicator of MPL biodegradation by the two microalgae. ER_t_/ER_0_ was plotted against the MPL residual fraction *f* according to Rayleigh equation, and linear fits with *R*^2^ of 0.53 and 0.65 were obtained (**Figure 2B** and **Supplementary Figure S3b**). The enantiomeric enrichment factors (ε_ER_) were 9.89% for *Haematococcus pluvialis* and 15.56% for *Scenedesmus quadricauda* given by the slope of linear regression line, indicating that their enantioselective biodegradation were slight and different between different microalgae species. Similarly, slight enantioselective biodegradation was observed during metoprolol degradation by activated sludge (Ribeiro et al., 2013). However, inconsistent results was also reported by Souchier et al. (2016), who found (S)-MPL enrichment during metoprolol degradation in laboratory water/sediment systems in dark conditions with ε_ER_ of −57% and suggested that enantiomeric fractionation was a good candidate for quantitative assessment of metoprolol biodegradation (Souchier et al., 2016). The above information indicated that the enantioselective behavior for the same compound could be different between microalgae and bacteria, and even among different microalgae species. It should be noted that compared with previous studies reporting strong linear fits with *R*^2^ above 0.9 (Jammer et al., 2014; Souchier et al., 2016), the *R*^2^ values in this study showed marginal linear correlation. Particularly, it seemed accelerated enrichment of (R)-MPL occurred in the last 5 days during MPL biodegradation by *Scenedesmus quadricauda* Breb (**Supplementary Figure S4**). Although this can be explained by Rayleigh equation that the more MPL was degraded, the more enriched (R)-MPL would be, the enrichment of (R)-MPL was still higher than the estimated value (**Supplementary Figure S3**). Therefore, considering that the previous studies (Jammer et al., 2014; Souchier et al., 2016) mostly focused on microorganisms in wastewater or pure enzymes from microorganisms and the understanding of enantioselective behavior of microalgae toward chiral contaminants is still limited, the less satisfying linear fits to Rayleigh equation implied that microalgae degradation could be more complicated and further research should be conducted to extrapolate applications of Rayleigh equation to other chiral contaminants and microalgae.

### Identification of TPs During MPL Degradation by Microalgae Using LC-HRMS/MS

**FIGURE 3:**
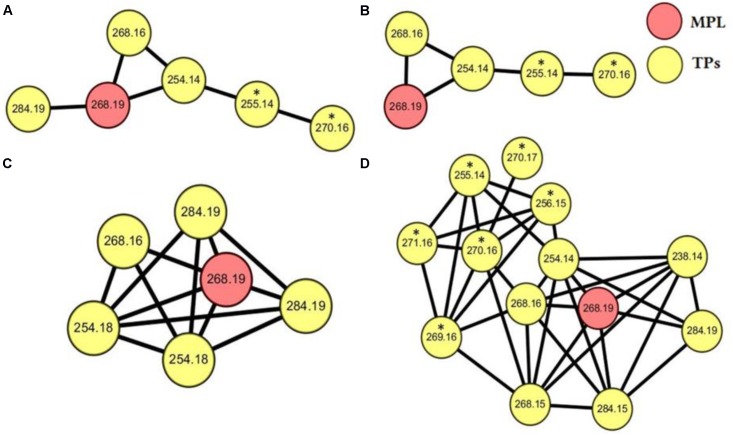
MS/MS spectral networking of MPL (red nodes) and its TPs (yellow nodes) for *Selenastrum capricornutum* **(A)**, *Chlorella vulgaris* **(B)**, *Scenedesmus quadricauda* Breb **(C)**, *Haematococcus pluvialis* **(D)**. The values listed in the nodes stand for the *m/z* of parent ions, and the parent ions which are directly connected by a line has MS/MS spectral with a cosine similarity above 0.6. The nodes annotated with asterisk **(^∗^)** represent the MS/MS of parent ions isolated from the isotope isomers.

**FIGURE 4:**
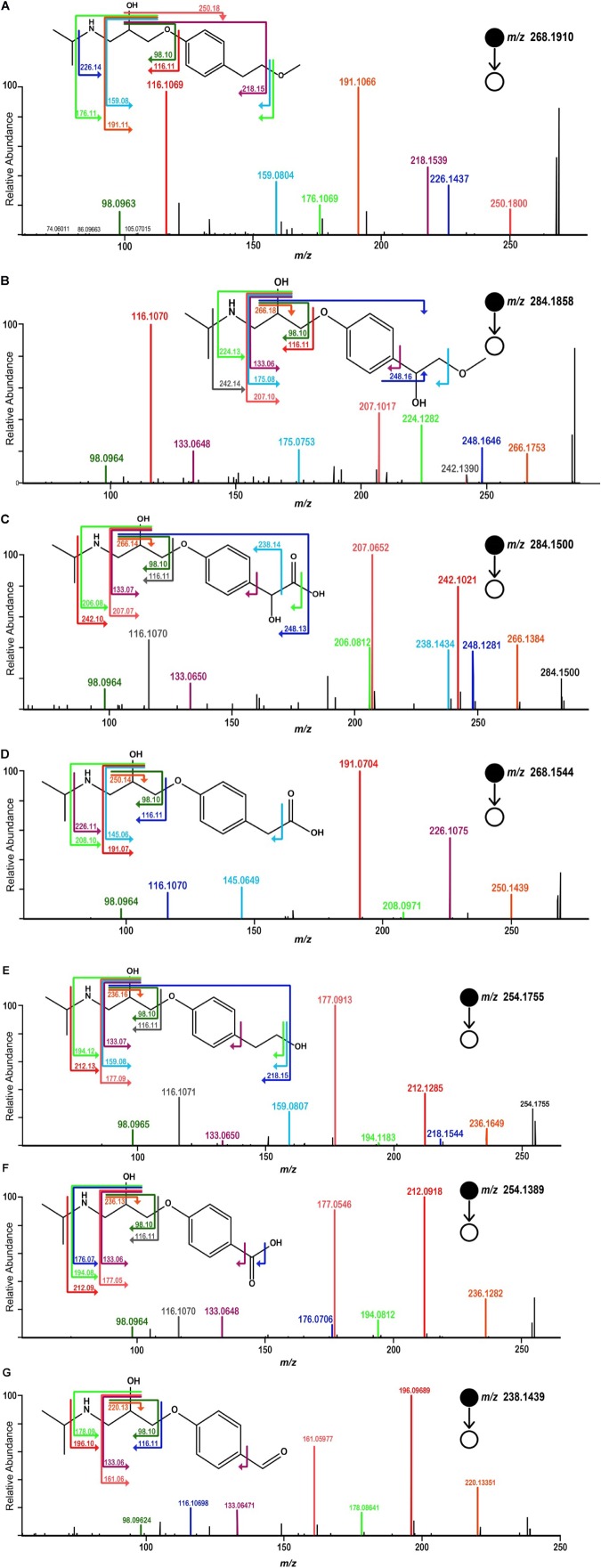
MS/MS spectra acquired by HRMS and the proposed fragmentation diagrams for [M + H]^+^ ion of MPL **(A)**, α-HMPL (*m/z* 284.1858) **(B)**, α-HMPLA (m/z 284.1500) **(C)**, MPLA (m/z 268.1544) **(D)**, O-DMPL (m/z 254.1755) **(E)**, DMPLA (m/z 254.1389) **(F)**, DMPLD (m/z 238.1439) **(G)**.

Samples, after SPE pretreatment, were analyzed by LC-HRMS/MS for all the four microalgae species. By applying MS/MS spectral similarity networking analysis, we found six compounds (*m/z* 284.1858, 284.1500, 268.1544, 254.1755, 254.1389, 238.1439) that were clustered with MPL (*m/z* 268.1910), implying that they are structurally similar (**Figure 3**). Their chemical structures were proposed based on the exact mass and ring and double bond equivalent (RDBE), giving the predicted chemical formulas to these compounds. The proposed chemical structures were further validated by their CID fragmentation products (**Figure 4** and **Supplementary Tables S2–S8**). Similar fragmentation pathways were observed for MPL and its TPs mainly with a neutral loss of H_2_O (18 Da), the loss of C_3_H_6_ (42 Da) and C_3_H_9_N (59 Da). Additionally, two fragment ions with *m/z* 98 and 116 were consistently detected in MPL and its TPs, confirming the consistent presence of the isopropyl portion of MPL. The postulated structures showed that the observed products were also chiral compounds, which was verified by the paired chromatographic peaks detected for all the TPs (**Supplementary Figure S5**). The TPs were named as α-hydroxymetoprolol (α-HMPL, *m/z* 284.1858), hydroxy{4-[2-hydroxy-3-(isopropylamino)propoxy]phenyl}acetic acid (α-HMPLA, *m/z* 284.1500), metoprolol acid (MPLA, *m/z* 268.1544), *O*-demethylmetoprolol (O-DMPL, *m/z* 254.1755), 4-[2-hydroxy-3-(isopropylamino)propoxy]benzoic acid (DMPLA, *m/z* 254.1389), and 4-[2-Hydroxy-3-(isopropylamino)propoxy]benzaldehyde (DMPLD, *m/z* 238.1439). Due to the lack of enantiopure standards of the TPs, the first and second eluting enantiomer was herein named E1 and E2, respectively. The detailed information regarding the TPs was presented in **Table 1**. All six chiral TPs were observed during MPL degradation by *Haematococcus pluvialis*, while α-HMPL, MPLA, and DMPLA were observed for all the four microalgae species. Additionally, O-DMPL was detected for *Scenedesmus quadricauda* Breb. Consistent results were found by MS/MS spectral similarity networking (**Figure 3**). The results demonstrated that MS/MS spectral similarity networking analysis could be a powerful tool to quickly identify TPs. It is worth mentioning that α-HMPL, DMPLA and O-DMPL were not included in **Figures 3B–D**, respectively, since their ion intensity were too low to be included in the detection list. Taking *m/z* 284.1858 (α-HMPL) as an example, it was less than 0.5% of the most intense peak as shown in **Supplementary Figure S6**, and it is very difficult to visually find out without intentionally extracting it. Therefore, it was not selected for subsequent fragment ion scan due to low intensity.

**Table 1:** Results of screening for TPs of MPL by LC-HRMS.

Compound name	Retention time (min)	Formula [M + H]^+^	Theoretical mass [M + H]^+^	Experimental mass [M+H]^+^	RDBE	Detected in microalgae species
(S)-MPL(R)-MPL	20.21 21.65	C_15_H_26_NO_3_	268.1907	268.1910	3.5	*Selenastrum capricornutum*, *Chlorella vulgaris*, *Scenedesmus quadricauda* Breb, *Haematococcus pluvialis*
(E1)-α-HMPL (E2)-α-HMPL	21.26 22.90	C_15_H_26_NO_4_	284.1856	284.1858	3.5	*Selenastrum capricornutum*, *Chlorella vulgaris*, *Scenedesmus quadricauda* Breb*, Haematococcus pluvialis*
(E1)-α-HMPLA	8.00	C_14_H_22_NO_5_	284.1493	284.1500	4.5	*Haematococcus pluvialis*
(E2)-α-HMPLA	8.93					
(E1)-MPLA (E2)-MPLA	8.67 9.62	C_14_H_22_NO_4_	268.1543	268.1544	4.5	*Selenastrum capricornutum*, *Chlorella vulgaris*, *Scenedesmus quadricauda* Breb, *Haematococcus pluvialis*
(E1)-O-DMPL (E2)-O-DMPL	20.82 22.33	C_14_H_24_NO_3_	254.1751	254.1755	3.5	*Scenedesmus quadricauda* Breb, *Haematococcus pluvialis*
(E1)-DMPLA	8.79	C_13_H_20_NO_4_	254.1387	254.1389	4.5	*Selenastrum capricornutum*,
(E2)-DMPLA	9.80					*Chlorella vulgaris*, *Scenedesmus quadricauda* Breb, *Haematococcus pluvialis*
(E1)-DMPLD	28.00	C_13_H_20_NO_3_	238.1438	238.1439	4.5	*Haematococcus pluvialis*
(E2)-DMPLD	29.60					

*RDBE stands for ring and double bond equivalent, obtained using Xcalibur software.*

**FIGURE 5:**
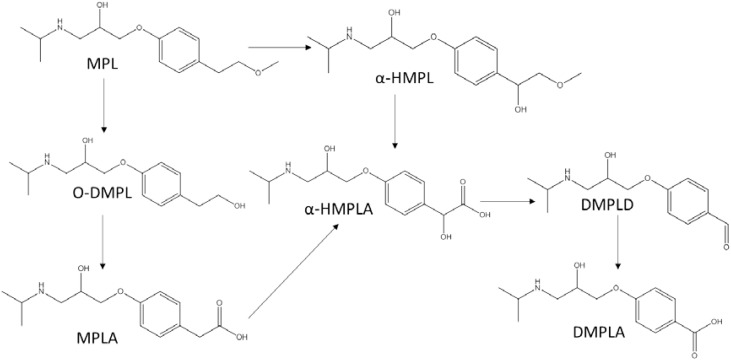
Proposed transformation pathways of MPL by microalgae species.

The proposed transformation pathway of MPL by *Haematococcus pluvialis* was given in **Figure 5**. Through α-hydroxylation and O-demethylation, MPL was accordingly transformed to α-HMPL and O-DMPL. O-DMPL was then oxidized into MPLA. α-HMPL, MPLA, and O-DMPL has been reported to be the three main metabolites of MPL in mammals (Murthy et al., 1990; Boralli et al., 2005). Recently, the occurrence and generation of the three metabolites were observed in activated sludge (Rubirola et al., 2014) and wetland microcosms (Svan et al., 2016). Other TPs including 4-(2-hydroxy-3-(isopropylamino)propoxy)phenol and 1-(4-(2-hydroxy-3-(isopropylamino)propoxy)phenyl)-2-methoxyethanone (Rubirola et al., 2014), as well as *N*-de-isopropylmetoprolol and deaminated metoprolol (Svan et al., 2016) were also reported; however, these four TPs were not detected in this study. Our study demonstrated that MPL could also be transformed into α-HMPL, MPLA, and O-DMPL by microalgae species such as *Scenedesmus quadricauda* Breb and *Haematococcus pluvialis*. Considering that O-DMPL showed slightly higher toxicity than MTP, α-HMPL and MPLA for *Vibrio fischeri* bioluminescent bacteria in an acute toxicity study (Rubirola et al., 2014), the generation of O-DMPL in this study implied that further studies should be conducted to assess its fate and risk. For *Selenastrum capricornutum* and *Chlorella vulgaris*, O-DMPL was not detected, implying that MPL was directly transformed to MPLA, or O-DMPL was not accumulated to be detected. Additionally, two new TPs of MPL including α-HMPLA, and DMPLD were firstly reported. Interestingly, although α-HMPLA and DMPLD were only simultaneously observed for *Haematococcus pluvialis*, DMPLA was detected for all the four microalgae species, suggesting that while different functional enzymes may be involved in MPL degradation among the four microalgae species, they generally oxidized MPL into α-HMPLA and MPLA, and then into DMPLA. Despite the discovery of the key role which MPLA played in MPL degradation, it has seldom been reported as a MPL intermediate in previous studies (Murthy et al., 1990; Boralli et al., 2005; Rubirola et al., 2014; Svan et al., 2016). A recent study in MPL degradation identified MPLA and DMPLA as TPs of MPL in soil, but no other TPs were found in the study (Koba et al., 2016). The above published results revealed that the degradation pathways for MPL still need to be further explored. Considering most studies concentrated on complex environment with mixed cultures, studies on MPL degradation by pure cultures would be strongly needed to better elucidate and understand the behavior of MPL in the environment.

## Conclusion

The targeted microalgae species were demonstrated to be able to simultaneously remove PO_4_^3−^- P, NO_3_^−^- N and MPL in the synthetic effluent, implying the potential of applying microalgae in wastewater treatment. Enantiomeric characterization was also conducted and stereoselectivity was observed during MPL degradation by the selected microalgae, with (S)-MPL being degraded faster than (R)-MPL. A marginal linear relationship between MPL degradation and enantiomeric enrichment implied that Rayleigh equation might be applied in microalgae degradation.

The degradation of the two enantiomers followed first order kinetics, and the identified TPs were all chiral and paired, implying that the two enantiomers could follow the same degradation mechanism for the selected microalgae. By using an efficient LC-HRMS/MS chiral analytical method and MS/MS spectral similarity networking, a total of 6 pairs of chiral TPs were identified for the four microalgae species, among which two new TPs of MPL including α-HMPLA, and DMPLD were reported for the first time, and transformation pathways of MPL by the microalgae were proposed. The results also demonstrated that MS/MS spectral similarity networking is a powerful tool in the identification of TPs.

## Author Contributions

C-PY conceived and designed the study and contributed to the editing and revision of this paper. ML performed the experiments and drafted the manuscript. CL, C-CH, and YW contributed to the experiments, data analysis, and revision of this paper. QS, YW, and Y-RC contributed to the experiments of this paper. YL and LC contributed to the revision of this paper. All the authors approved it for publication.

## Conflict of Interest Statement

The authors declare that the research was conducted in the absence of any commercial or financial relationships that could be construed as a potential conflict of interest.

## Footnotes

1 http://gnps.ucsd.edu
