# Supplementary material for: Identification of Enantiomeric Byproducts During Microalgae-Mediated Transformation of Metoprolol by MS/MS Spectrum Based Networking
Source: Front Microbiol. 2018 Sep 7;9:2115. doi: 10.3389/fmicb.2018.02115 (PMC6137207; doi:10.3389/fmicb.2018.02115)
Supplement: Supplementary file 1 [file Table_1.DOCX]

**Supplemental Information**

**Identification of Enantiomeric Byproducts During Microalgae-mediated Transformation of Metoprolol by MS/MS Spectrum Based Networking**

Min Lv^1,2^, Ching Lo^3^, Cheng-Chih Hsu^3^, Yuwen Wang^4^, Yin-Ru Chiang^5^, Qian Sun^4^, Yang Wu^4^, Yan Li^4^, Lingxin Chen^1^, Chang-Ping Yu^2*^

^1^CAS Key Laboratory of Coastal Environmental Processes and Ecological Remediation, Yantai Institute of Coastal Zone Research, Chinese Academy of Sciences, Yantai 264003, China

^2^Graduate Institute of Environmental Engineering, National Taiwan University, Taipei 10617, Taiwan

^3^Department of Chemistry, National Taiwan University, Taipei 10617, Taiwan

^4^CAS Key Laboratory of Urban Pollutant Conversion, Institute of Urban Environment, Chinese Academy of Sciences, Xiamen 361021, China

^5^Biodiversity Research Center, Academia Sinica, Taipei, Taiwan

* Corresponding Author:

Dr. Chang-Ping Yu, Tel: 886-2-3366-3729, Email: cpyu@ntu.edu.tw

**Supplemental Information**

24 pages, including 8 tables and 6 figures.

Page 3 provides details on the sampling and analytical methods.

Pages 4 includes Table S1, which is referred to directly in the Materials and methods portion.

Pages 5-10 include Figure S1-S6, which are referred to directly in the Results and discussion portion of the text.

Pages 11-24 include Table S2-S8, which are the detailed MS/MS peaks annotation of MPL standard and TPs.

**Material and methods**

**Analysis of water quality parameters**

Samples were ﬁltered through 0.45 μm pore-size cellulose ﬁlters (Millipore, Billerica, MA, USA) and frozen until analysis within a week. PO_4_^3-^- P in the ﬁltrate was processed following an alkaline potassium persulfate digestion. Ammonium (NH_4_^+^), nitrate (NO_3_^-^ -N) in the filtrate as well as PO_4_^3-^- P in the digest were analyzed by a ﬂow injection analyzer (QC8500, Lachat^®^, Loveland, Co., USA).

**Sample preparation for identification of transformation products**

Oasis HLB cartridge (500 mg, 6 mL, Waters) were preconditioned with 20 mL methanol, 6 mL water, and 6 mL water under pH 2.0. The samples were passed through the cartridges at a flow rate around 3 mL/min. After the cartridges were dried under an airstream for 10 min, analytes were eluted with 12 mL of methanol and 6 mL of acetone-methanol 50/50 (v/v). The elutes were collected, evaporated to dryness with nitrogen gas and reconstituted to 1.0 mL with methanol.

**TABLE S1**│ Component of synthetic wastewater for MPL degradation

| Compound | Concentration (mM) | Compound (trace metal) | Concentration (mM) |
| --- | --- | --- | --- |
| NaNO_3_ | 0.86 | FeCl_2_.4H_2_O | Fe^2+^ = 0.01 |
| K_2_HPO_4_ | 0.19 | MnSO_4_.H_2_O | Mn^2+^ = 0.02 |
| NaCl | 0.43 | (NH_4_)_6_ Mo_7_O_24_.4H_2_O | Mo_7_O_24_^6-^ = 0.001 |
| NaHCO_3_ | 1 |  | NH_4_^+^ = 0.006 |
| MgSO_4_ | 0.3 | CuSO_4_ | Cu^2+^ = 0.005 |
| CaCl_2_.2H_2_O | 0.17 | ZnSO_4_.7H_2_O | Zn^2+^ = 0.006 |
| EDTA disodium salt | 0.01 | NiSO_4_.6H_2_O | Ni^2+^ = 0.001 |

**FIGURE S1│** Liquid chromatogram for a 20 d sample during metoprolol degradation by *Haematococcus pluvialis*.

**FIGURE S2**│ The removal of nutrient and metoprolol by *Selenastrum capricornutum* and *Chlorella vulgaris* and their growth in the synthetic effluent. The MPL concentrations were the sum of (S)-MPL and (R)-MPL concentrations.

**FIGURE S3│** (S)-MPL and (R)-MPL concentrations changes versus time during MPL biodegradation by *Scenedesmus quadricauda* Breb (a) and Rayleigh representation of the enantiomeric enrichment of MPL versus MPL biodegradation (b). Insets: Kinetics of first-order biodegradation of the two MPL enantiomers.

**FIGURE S4│** EF changes against time during MPL biodegradation by *Haematococcus pluvialis* and *Scenedesmus quadricauda* Breb.

(a)

(b)

(e)

(d)

(c)

(f)

**FIGURE S5│** LC-HRMS chromatogram of six pairs of transformation products with (a), (b), (c), (d), (e), (f) representing chiral DMPLD, DMPLA, O-DMPL, MPLA, α-HMPLA, α-HMPL, respectively. The extracted chromatogram of the control is shown in the upper window of (a), (b), (c), (d), (e) and (f).

**FIGURE S6│** The HRMS spectrum corresponding to extracted chromatogram peak of [M+H]^+^ of 284.1858 Da during degradation of MPL by *Chlorella vulgaris*.

**TABLE S2**│ Fragmentation structures of **MPL**

| Structure | Exact mass | Experiment | Error(ppm) |
| --- | --- | --- | --- |
| [M+H]^+^ **(­parent ion)**   | 268.1907 | 268.1910 | 1.12 |
| [M-H_2_O+H]^+^   | 250.1802 | 250.1800 | -0.80 |
| [M-C3H7+H+H]^+^   | 226.1438 | 226.1437 | -0.44 |
| [M-H_2_O-CH_3_OH+H]^+^   | 218.1539 | 218.1539 | -0.09 |
| [M-C_3_H_6_-CH_3_OH+H]^+^   | 194.1176 | 194.1174 | -1.03 |
| [M-H_2_O-C_3_H_7_NH]^+^   | 191.1067 | 191.1066 | -0.52 |
| Structure | Exact mass | Experiment | Error(ppm) |
| [M-H_2_O-C_3_H_6_-CH_3_OH+H]^+^   | 176.1070 | 176.1069 | -0.57 |
| [M-H_2_O-CH_3_OH-C_3_H_7_NH]^+^   | 159.0804 | 159.0804 | -0.57 |
| [M-CH_3_OC_2_H_4_C_6_H_4_OH+H]^+^   | 116.1070 | 116.1069 | -0.86 |
| [M- CH_3_OC_2_H_4_C_6_H_4_O-H_2_O]^+^   | 98.0964 | 98.0963 | -1.02 |

**TABLE S3**│ Fragmentation structures of **α-HMPL**

| Structure | Exact mass | Experiment | Error(ppm) |
| --- | --- | --- | --- |
| [M+H]^+^ **(­parent ion)**   | 284.1856 | 284.1858 | 0.70 |
| [M-H_2_O+H]^+^   | 266.1751 | 266.1753 | 0.75 |
| [M-2H_2_O+H]^+^   | 248.1645 | 248.1646 | 0.40 |
| [M-C_3_H_7_+H+H]^+^   | 242.1387 | 242.1390 | 1.24 |
| [M-H_2_O -C_3_H_7_ +H+H]^+^   | 224.1281 | 224.1282 | 0.45 |
| [M-H_2_O-C_3_H_7_NH]^+^   | 207.1016 | 207.1017 | 0.48 |
| Structure | Exact mass | Experiment | Error(ppm) |
| [M-H_2_O-C_3_H_7_NH-CH_3_OH]^+^   | 175.0754 | 175.0753 | -0.57 |
| [M-H2O-C_3_H_7_NH-CH_3_OCH_2_CHOH+H]^+^   | 133.0648 | 133.0648 | 0.00 |
| [M-CH_3_OCH_2_CHOHC_6_H_4_OH+H]^+^   | 116.1070 | 116.1070 | 0.00 |
| [M-CH_3_OCH_2_CHOHC_6_H_4_O-H_2_O]^+^   | 98.0964 | 98.0964 | -0.41 |

**TABLE S4**│ Fragmentation structures of **α-HMPLA**

| Structure | Exact mass | Experiment | Error(ppm) |
| --- | --- | --- | --- |
| [M+H]^+^ **(­parent ion)**   | 284.1493 | 284.1494 | 0.35 |
| [M-H_2_O+H]^+^   | 266.1387 | 266.1384 | -1.13 |
| [M-2H_2_O+H]^+^   | 248.1281 | 248.1281 | -0.20 |
| [M-C_3_H_7_+H+H]^+^   | 242.1023 | 242.1021 | -0.83 |
| [M-CO_2_H]^+^   | 238.1438 | 238.1434 | -1.68 |
| Structure | Exact mass | Experiment | Error(ppm) |
| [M-H_2_O-C_3_H_7_NH]^+^   | 207.0652 | 207.0652 | 0.19 |
| [M-2H_2_O-C_3_H_7_+H+H]   | 206.0812 | 206.0812 | 0.24 |
| [M-H2O-C_3_H_7_NH-CO_2_HCHOH+H]^+^   | 133.0648 | 133.0650 | 1.50 |
| [M-CO_2_HCHOHC_6_H_4_OH+H]^+^   | 116.1070 | 116.1070 | 0.00 |
| [M-H_2_O-CO_2_HCHOHC_6_H_4_O]^+^   | 98.0964 | 98.0964 | -0.20 |

**TABLE S5**│ Fragmentation structures of **MPLA**

| Structure | Exact mass | Experiment | Error(ppm) |
| --- | --- | --- | --- |
| [M+H]^+^ **(­parent ion)**   | 268.1543 | 268.1544 | 0.37 |
| [M-H_2_O+H]^+^   | 250.1438 | 250.1439 | 0.40 |
| [M-C3H7+H+H]^+^   | 226.1074 | 226.1075 | 0.44 |
| [M-H_2_O-C_3_H_7_+H+H]^+^   | 208.0968 | 208.0971 | 1.44 |
| [M-H_2_O-C_3_H_7_NH]^+^   | 191.0703 | 191.0704 | 0.53 |
| [M-H_2_O-C_3_H_7_NH_2_-CO_2_H]^+^   | 145.0648 | 145.0649 | 0.69 |
| Structure | Exact mass | Experiment | Error(ppm) |
| [M-CO_2_HCH_2_C_6_H_4_OH+H]^+^   | 116.1070 | 116.1070 | 0.00 |
| [M-H_2_O -CO_2_HCH_2_C_6_H_4_O]^+^   | 98.0964 | 98.0964 | -0.20 |

**TABLE S6**│ Fragmentation structures of **O-DMPL**

| Structure | Exact mass | Experiment | Error(ppm) |
| --- | --- | --- | --- |
| [M+H]^+^ **(­parent ion)**   | 254.1751 | 254.1753 | 0.79 |
| [M-H_2_O+H]^+^   | 236.1645 | 236.1649 | 1.69 |
| [M-2H_2_O+H]^+^   | 218.1539 | 218.1543 | 1.83 |
| [M-C_3_H_7_+H+H]^+^   | 212.1281 | 212.1285 | 1.88 |
| [M-C_3_H_7_-H_2_O+H]^+^   | 194.1176 | 194.1183 | 3.61 |
| [M-H_2_O-C_3_H_7_NH]^+^   | 177.0910 | 177.0913 | 1.69 |
| Structure | Exact mass | Experiment | Error(ppm) |
| [M-2H_2_O-C_3_H_6_+H]^+^   | 176.1070 | 176.1073 | 1.70 |
| [M-2H_2_O-C_3_H_7_NH]^+^   | 159.0804 | 159.0807 | 1.89 |
| [M-H_2_O-CH_2_OHCH_2_-C_3_H_7_NH+H]^+^   | 133.0648 | 133.0650 | 1.50 |
| [M-CH_2_OHCH_2_C_6_H_4_OH+H]^+^   | 116.1070 | 116.1071 | 0.86 |
| [M-H_2_O-CH_2_OHCH_2_C_6_H_4_O]^+^   | 98.0964 | 98.0963 | -1.02 |

**TABLE S7**│ Fragmentation structures of **DMPLA**

| Structure | Exact mass | Experiment | Error(ppm) |
| --- | --- | --- | --- |
| [M+H]^+^ **(­parent ion)**   | 254.1387 | 254.1389 | 0.79 |
| [M-H_2_O+H]^+^   | 236.1281 | 236.1282 | 0.42 |
| [M-C_3_H_7_+H+H]^+^   | 212.0917 | 212.0918 | 0.47 |
| [M-H_2_O-C_3_H_7_+H+H]^+^   | 194.0812 | 194.0812 | 0.05 |
| [M-H_2_O-C_3_H_7_NH]^+^   | 177.0546 | 177.0546 | 0.06 |
| Structure | Exact mass | Experiment | Error(ppm) |
| [M-H_2_O-C_3_H_6_-OH]^+^   | 176.0706 | 176.0706 | 0.00 |
| [M-H_2_O-CO_2_-C_3_H_7_NH]^+^   | 133.0648 | 133.0648 | 0.23 |
| [M-CO_2_HC_6_H_4_OH+H]^+^   | 116.1070 | 116.1070 | -0.09 |
| [M-H_2_O-CO_2_HC_6_H_4_O]^+^   | 98.0964 | 98.0964 | -0.41 |

**TABLE S8**│ Fragmentation structures of **DMPLD**

| Structure | Exact mass | Experiment | Error(ppm) |
| --- | --- | --- | --- |
| [M+H]^+^ **(­parent ion)**   | 238.1438 | 238.1439 | 0.37 |
| [M-H_2_O+H]^+^   | 220.1332 | 220.1335 | 1.36 |
| [M-C_3_H_7_+H+H]^+^   | 196.0968 | 196.0969 | 0.51 |
| [M-H_2_O-C_3_H_7_+H+H]^+.^   | 178.0863 | 178.0864 | 0.56 |
| [M-H_2_O -C_3_H_7_NH]^+^   | 161.0597 | 161.0598 | 0.62 |
| [M-H_2_O-CO_2_-C_3_H_7_NH]^+^   | 133.0648 | 133.0647 | -0.75 |
| Structure | Exact mass | Experiment | Error(ppm) |
| [M-CH_2_OC_6_H_4_OH+H]^+^   | 116.1070 | 116.1070 | -0.06 |
| [M-H_2_O-CH_2_OC_6_H_4_O]^+^   | 98.0964 | 98.0962 | -2.04 |
